# Midazolam Boosting With Cobicistat in a Patient With Drug-Resistant Epilepsy and Focal Status Epilepticus

**DOI:** 10.1097/FTD.0000000000001283

**Published:** 2024-11-20

**Authors:** Tessa van den Born-Bondt, Niels Westra, Katarzyna Krzywicka, Harmen R. Moes, Manon Schuls-Fouchier, Daan J. Touw, Thijs H. Oude Munnink

**Affiliations:** * Department of Clinical Pharmacy and Pharmacology, University Medical Center Groningen; and; † Department of Neurology, University Medical Center Groningen, Groningen, the Netherlands.

**Keywords:** pharmacokinetic boosting, pharmacokinetic enhancement, cobicistat, midazolam, clobazam, epilepsy

## Abstract

**Background:**

This report presents the case of a patient with drug-resistant epilepsy. Despite treatment with 4 antiepileptic drugs, the patient experienced an increasing frequency of focal seizures, necessitating hospitalization, and continuous intravenous midazolam infusion.

**Methods:**

Cobicistat was introduced as a pharmacokinetic booster to decrease the metabolic clearance of midazolam, leading to increased exposure and an extended half-life.

**Results:**

Cobicistat boosting allowed the switch from intravenous to oral midazolam, and the patient was discharged on an oral midazolam regimen.

**Conclusions:**

Cobicistat can be effectively used to boost midazolam exposure pharmacokinetically in patients with drug-resistant epilepsy who require stable midazolam blood concentrations.

## CLINICIAN

A 29-year-old male patient with left-sided hippocampal sclerosis and drug-resistant mesial temporal lobe epilepsy, ineligible for epilepsy surgery, was hospitalized for focal status epilepticus, experiencing up to 80 focal seizures per day. Seizures are typically manifested as tonic-clonic movements of the right arm with impaired awareness, lasting 20–60 seconds. His medical history revealed multiple recent hospitalizations for focal status epilepticus, with durations up to 100 days because of difficulties in tapering intravenous midazolam. On admission, antiepileptic drugs included clobazam 15 mg three times daily, lamotrigine 225 mg two times daily, levetiracetam 1000 mg three times daily, midazolam 7.5 mg three times daily, and 11.25 mg at night, and rescue midazolam nasal spray PRN (5 mg). Other medications included esomeprazole, cholecalciferol, and famotidine.

Oral midazolam was replaced with intravenous midazolam 7.9 mg/h (0.1 mg/kg/h), after which the seizures stopped. The plan was to reduce intravenous midazolam by 1.0 mg/h every 3 days. Over 3 weeks, intravenous midazolam was reduced to 2.0 mg/h, whereas oral midazolam was reintroduced at 7.5 mg three times daily and 11.25 mg at night. Seizures increased when the intravenous midazolam dose was further reduced to 1.0 mg/h. Intravenous midazolam was reverted to 2.0 mg/h, with 7.5-mg midazolam orally two times daily and 11.25 mg at night to achieve acceptable seizure control.

Continuous intravenous midazolam administration could not be tapered, preventing patient discharge. Is there a strategy for mimicking continuous midazolam exposure using an oral midazolam regimen?

## TDM CONSULTANT

A midazolam half-life of 1.5–2.5 hours^1,2^ necessitates a short interval between oral administrations to achieve stable exposure. Cobicistat, a potent CYP3A4 inhibitor, can boost midazolam exposure. Cobicistat increases midazolam AUC 11- to 12-fold and half-life to 11–19 hours.^3^ Cobicistat-boosted midazolam likely best mimics the exposure profile of continuous infusion. Measurement of midazolam baseline concentrations is necessary because this concentration currently provides adequate seizure control.

## CLINICIAN

Midazolam baseline concentrations over the past 5 days were 76 and 90 mcg/L. For this patient, we accept up to 3 focal seizures per day, whereas generalized seizures are acceptable up to once a week.

## TDM CONSULTANT

Baseline midazolam concentrations were within the expected steady-state range for the current midazolam dose of 130 mg daily oral equivalent, considering an average bioavailability of 50% and a volume of distribution of 1 L/kg.^1,2^ This indicates normal CYP3A4 activity. The cobicistat-boosted midazolam regimen may be assumed to have an estimated 100% bioavailability as a worst-case scenario, assuming complete CYP3A4 inhibition (including intestinal CYP3A4) and a boosted half-life of 18 hours.^3^ The estimated equivalent daily dose was approximately 6-mg midazolam when boosted with cobicistat. NONMEM simulations, based on a midazolam population pharmacokinetic model with a strong CYP3A4 inhibitor as a covariate, confirmed the estimated dosing regimen.^4^ NONMEM simulations indicated that midazolam 3.75 mg two times daily boosted with cobicistat 150 mg once daily will result in an exposure equivalent to the baseline. Discontinue intravenous midazolam infusion one hour after switching to cobicistat-boosted oral midazolam. In addition, use nasal midazolam escape at 0.5 mg. Furthermore, a 50% dose reduction is recommended for clobazam because it is primarily metabolized by CYP3A4.^5–7^

## CLINICIAN

**FIGURE 1.**
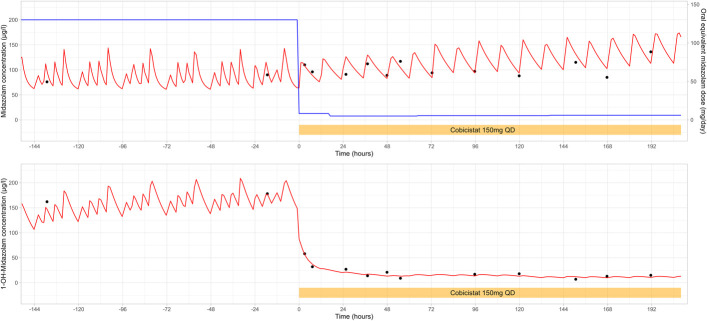
Measured and simulated midazolam (mcg/L, upper panel) and 1-hydroxymidazolam (mcg/L, lower panel) concentrations over time. The red line represents the NONMEM-simulated midazolam and 1-hydroxymidazolam concentrations. The black dots represent the observed/measured concentrations. The blue line, with the corresponding right axis, represents the oral equivalent daily dose of midazolam (mg/d). The cobicistat-boosted oral midazolam regimen was initiated at t = 0 hours, as indicated by the orange rectangular region.

As recommended, cobicistat 150 mg once daily was initiated, and the midazolam infusion was switched to oral midazolam 3.75 mg two times daily. As a precautionary measure, the patient is observed in the neurocritical care unit with continuous monitoring of vital signs. The patient experienced increased somnolence after the first dose of midazolam without signs of respiratory depression. Midazolam concentrations at 2 and 6 hours after dose were 116 mcg/L and 96 mcg/L, respectively, with a decline in 1-hydroxymidazolam concentrations (Fig. 1).

## TDM CONSULTANT

The measured midazolam and 1-hydroxymidazolam concentrations demonstrated the immediate boosting effect of cobicistat on midazolam pharmacokinetics, resulting in a prolonged half-life. Figure 1 shows comparable midazolam concentrations before and after cobicistat introduction, despite the significantly reduced midazolam dose. The 1-hydroxymidazolam concentrations decreased because of the inhibition of CYP3A4-mediated metabolism of midazolam. Daytime somnolence may indicate higher than necessary exposure. NONMEM simulated trough and peak concentrations for 3.75 mg two times daily are 96 mcg/L and 140 mcg/L. The recommended dose was adjusted to 2.5-mg oral midazolam two times daily. Expected trough and peak concentrations with this dose are 80 mcg/L and 110 mcg/L. The midazolam concentration was measured the next day to monitor the effects of dose adjustment.

## CLINICIAN

The midazolam dose was adjusted to 2.5 mg two times daily, with peak concentrations of 91 mcg/L in the morning and 112 mcg/L in the evening. The patient is less sleepy. He experienced a few focal seizures, which are within the range of what is considered acceptable.

## TDM CONSULTANT

Midazolam concentrations were as expected. Midazolam nasal spray has not yet been administered. We recommend testing midazolam nasal spray when seizures occur to ensure that a 0.5-mg nasal spray is an adequate emergency treatment in the outpatient setting. Measurement of trough and peak concentrations around the time of nasal spray administration is advised.

## CLINICIAN

The patient received a midazolam nasal spray (0.5 mg) between two oral doses of 2.5 mg. Trough and peak concentrations were 117 mcg/L and 127 mcg/L, respectively. Nasal midazolam was well-tolerated, causing only brief dizziness and no other side effects.

## TDM CONSULTANT

Given the minor side effects of nasal midazolam and the ongoing occurrence of minor focal seizures, the midazolam dose was increased to 3 mg in the morning and 2.5 mg in the evening. Trough concentrations were measured daily to monitor the impact of the adjustment.

## CLINICIAN

Midazolam trough concentrations over the past 3 days were 94 mcg/L, 97 mcg/L, and 88 mcg/L, respectively. The number of seizures was within the acceptable range but varied between days.

## TDM CONSULTANT

Despite steady blood concentrations, a small number of focal seizures persisted. Increase the midazolam dose to 3 mg two times daily. The clobazam trough concentration was measured to ensure it remained comparable to the precobicistat concentration.

## CLINICIAN

The midazolam dose was adjusted to 3 mg two times daily, resulting in a midazolam trough concentration of 136 mcg/L. The clobazam trough concentration was 703 mcg/L. The number of seizures was within the acceptable range.

## TDM CONSULTANT

The blood concentrations of clobazam and midazolam were stable and within the expected range. Clobazam exposure at the 50% reduced dose was comparable to the precobicistat exposure; therefore, no further dose adjustments were required. Further measurement of blood concentrations is unnecessary. An emergency protocol was established in case of readmission and shared with all healthcare providers involved in the hospital and primary care settings.

## FOLLOW-UP

The patient was discharged 10 days after starting cobicistat treatment, experiencing an acceptable number of seizures. Three weeks after discharge, a telephone consultation revealed one major seizure, which was successfully treated with nasal midazolam (0.5 mg). This incident did not exceed the established seizure acceptance criteria. The patient continued to have focal seizures, which were considered acceptable. Five weeks after discharge, the patient was readmitted to the hospital because of an increased seizure frequency. On readmission, cobicistat was continued, and seizure control was achieved with a continuous intravenous infusion of midazolam at a rate of 0.6 mg/h, whereas oral midazolam was discontinued. After a few days, the midazolam infusion was successfully switched to oral midazolam at a dose of 3.75 mg four times daily, and the patient was discharged. No further hospitalizations were necessary, and the patient is now >6 months on the cobicistat-boosted regimen with an acceptable quality of life.
